# Differential gene analysis during the development of obliterative bronchiolitis in a murine orthotopic lung transplantation model: A comprehensive transcriptome-based analysis

**DOI:** 10.1371/journal.pone.0232884

**Published:** 2020-05-08

**Authors:** Atsushi Hata, Hidemi Suzuki, Takahiro Nakajima, Taiki Fujiwara, Yuki Shiina, Taisuke Kaiho, Takahide Toyoda, Terunaga Inage, Takamasa Ito, Yuichi Sakairi, Hajime Tamura, Hironobu Wada, Yoshito Yamada, Masako Chiyo, Keisuke Matsusaka, Masaki Fukuyo, Ken-ichi Shinohara, Sakae Itoga, Shinichiro Motohashi, Kazuyuki Matsushita, Atsushi Kaneda, Ichiro Yoshino

**Affiliations:** 1 Department of General Thoracic Surgery, Chiba University Graduate School of Medicine, Chiba, Japan; 2 Department of Medical Immunology, Chiba University Graduate School of Medicine, Chiba, Japan; 3 Department of Molecular Oncology, Chiba University Graduate School of Medicine, Chiba, Japan; 4 Department of Genome Research and Development, Kazusa DNA Research Institute, Chiba, Japan; 5 Department of Laboratory Medicine & Division of Clinical Genetics and Proteomics, Chiba University Graduate School of Medicine, Chiba, Japan; University of Kentucky, UNITED STATES

## Abstract

**Background:**

Obliterative bronchiolitis (OB) is a known issue during minor histocompatibility antigen (mHA) disparity during lung transplantation. This study evaluated gene expression in a murine orthotropic lung transplantation model using microarray analysis.

**Methods:**

Left lungs from C57BL/10(H-2^b^) donor mice were transplanted into mHA-mismatched C57BL/6(H-2^b^) recipient mice. Three groups (OB, non-OB, and sham controls) were confirmed pathologically and analyzed. Gene expression changes in the lung grafts were determined by microarray and immunohistochemical staining, and genes were verified by quantitative PCR in the lungs and mediastinal lymph nodes (LNs).

**Results:**

A total of 1343 genes were upregulated in the OB lungs compared to the sham group. Significant upregulation was observed for genes related to innate, e.g. *Tlr2 and CCL3* and adaptive immunity, e.g. *H2-ab1* and *Il-21*. Positive labeling for MHC class II antigen was observed in the bronchial epithelium of OB accompanied with B cells. We found increased *Tlr2*, *Ccl3*, *H2-ab1*, *Il-21*, *Ighg3*, *Ifng*, and *Pdcd1* mRNA expression in the OB lung, and increased *Il-21*, *Ighg3*, and *Pdcd1* expression in the OB LNs.

**Conclusions:**

Adaptive and innate immune reactions were involved in OB after lung transplantation, and genetic examination of related genes could be used for detection of OB.

## Introduction

Bronchiolitis obliterans syndrome (BOS), a major form of chronic lung allograft dysfunction, has the greatest impact on the long-term survival of lung transplant patients [1, 2]. Various pathologies have been associated with BOS, including innate immune system activation, acute cellular rejection, autoimmunity, and antibody-mediated rejection (AMR) via donor specific antibodies [3–7]. Current therapeutic options, such as macrolide antibiotic treatment and immunosuppressants, have been shown to stabilize pulmonary function [2, 8, 9]. Unfortunately, despite numerous studies concerning the development, progression, and treatment of BOS, the detailed mechanisms underlying pathogenesis have not been fully elucidated.

BOS is characterized by obliterative bronchiolitis (OB), or the fibrous remodeling of the small peripheral airways [10]. However, OB distribution is patchy and discontinuous making the histological detection difficult using traditional transbronchial biopsies (TBBs) [5, 11, 12]. Comprehensive gene expression analysis using microarray technology has also recently been conducted to detect OB [13–16]. Of these studies, two analyzed human bronchoalveolar lavage samples [14, 15], while the another focused on murine heterotopic trachea transplants [13, 17]. Only one report described OB detection in a rat orthotopic lung transplantation model [16].

Recently, we developed a murine OB orthotopic lung transplantation model [18]. Briefly, when lungs from C57BL/10(H2^b^) mice were transplanted orthotopically into minor histocompatibility antigen (mHA)-mismatched C57BL/6(H2^b^) mice, OB occurred by day 21 post-transplantation in approximately 50% of the recipients [19]. This and other orthotopic lung transplantation models are ideal for evaluating clinical situations. Their use is essential to understand the mechanisms underlying OB pathogenesis.

In this study, we investigated OB in our recently established murine orthotopic lung transplantation model using microarray analysis to evaluate mRNA expression changes and to search for novel disease biomarkers. As this model is new, the present study provides important insight into OB progression after lung transplantation.

## Materials and methods

**Fig 1:**
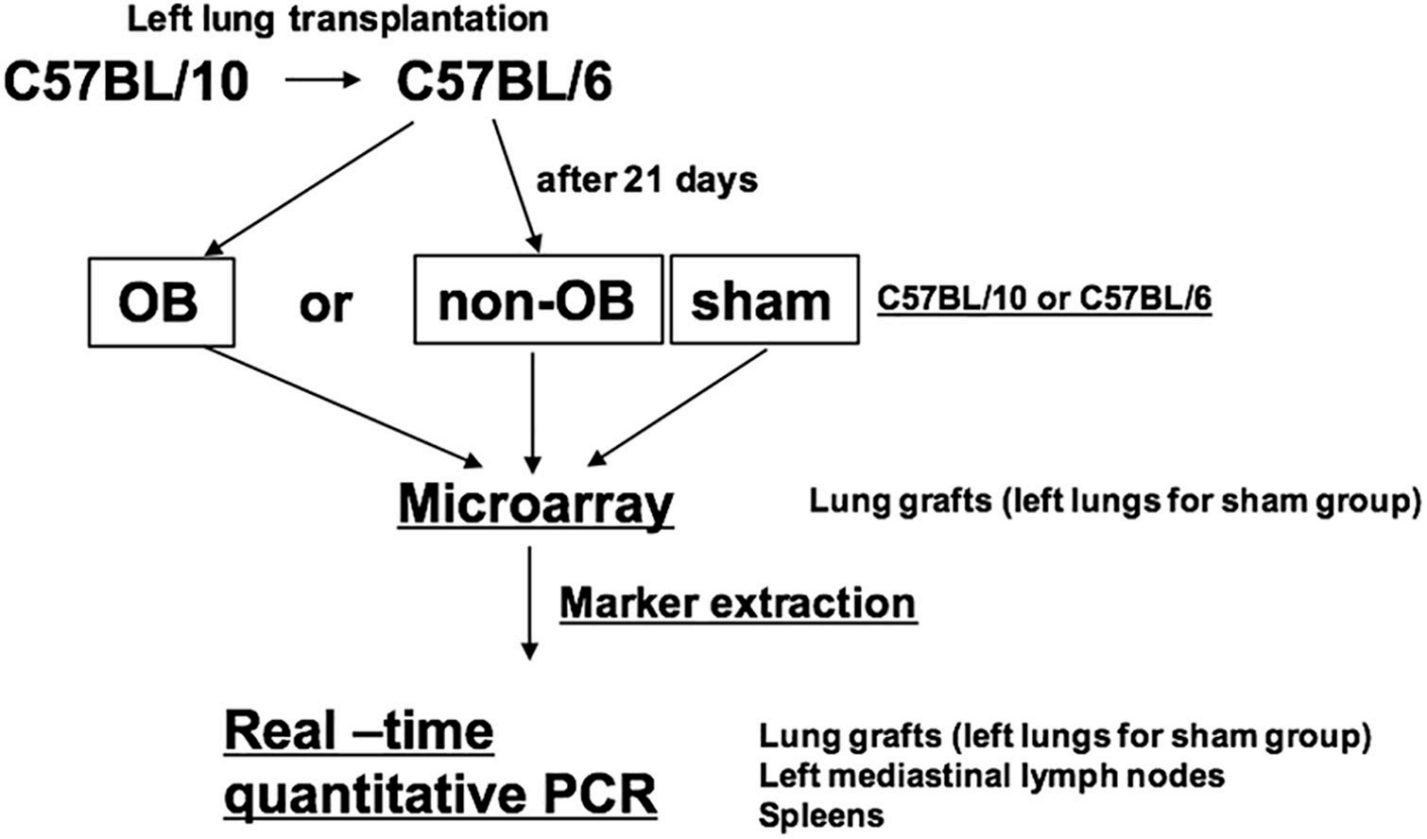
Study design. Left lung from C57BL/10 inbred mice were orthotopically transplanted into minor histocompatibility antigen mismatched C57BL/6 mice. Both inbred mice were used for sham. The transplanted lung grafts were harvested and classified into OB or non-OB by pathological examination at 21th day post transplantation. The left lungs of shams were also harvested at the same time. Three samples in each group (total 9 lungs) were subjected individually to microarray analysis. After representative OB marker genes were extracted from microarray analysis, real-time quantitative PCR were performed for lungs, left mediastinal lymph nodes, and spleens using at least 5 animals for each group.

Detailed information regarding the methods used in this study is provided in the Supplemental Material and Methods. The study design is shown in Fig 1.

### Animals

Specific pathogen-free male inbred C57BL/6 (H2^b^) and C57BL/10(H2^b^) mice were purchased from CLEA Japan, Inc. (Tokyo, Japan) and the Central Institute for Experimental Animals (Kanagawa, Japan), respectively. They were housed at the Biomedical Research Center at Chiba University School of Medicine in accordance with institutional guidelines. C57BL/10 (H2^b^) were used as donors and C57BL/6(H2^b^) as recipients at 8–12 weeks of age (body weight, 24–32 g). Inbred C57BL/6(H2^b^) and C57BL/10(H2^b^) mice were housed at the Biomedical Research Center at Chiba University School of Medicine in accordance with institutional guidelines. Both strains were used as donors and/or recipients.

### Surgical technique

Orthotopic transplantation was performed as previously described [18] and also described in Suppremental Materials and Methods. C57BL/10 (H2^b^) were used as donors and C57BL/6(H2^b^) as recipients and only thoracotomy was performed for the sham group. C57BL/10 and C57BL/6 are used for sham group. The transplanted lung grafts and the left lungs in the sham group were harvested on day 21 post-surgery along with mediastinal lymph nodes (LNs) and spleens. This study was approved by the Institute for Animal Care at Chiba University (approval code: A29-103) and was performed in compliance with the Guide for the Care and Use of Laboratory Animals (National Institutes of Health publication 86–23, revised 1996). All researchers engaged in the animal procedure take institutional annual training for animal care and handling. All surgical procedures were performed utilizing sterile technique. No antibiotics are given to both donor and recipient mice. Induction of anesthesia of the donor mouse is initiated with 5% Isoflurane. The mouse is orotracheally intubated with a 20-gauge intravenous catheter and then placed on a rodent ventilator, using 100% oxygen at rate of 125 breaths/minute and approximately 0.5 ml tidal volume (2% of its body weight). The animals are maintained under general anesthesia with a mixture of isoflurane and oxygen. The donor and recipient mice are placed prepped with 70% alcohol before incision. Buprenorphine (0.05–0.15 mg/kg) was administered immediately after surgery and every 8 hours for 2–3 days post-surgery to reduce pain and all efforts were made to minimize suffering. Animals will be monitored every day for signs of respiratory difficulty, weight loss, any development of a moribund state. Greater than 15% weight loss from pre-operative weights, lack of food and/or water consumption for more than 48 hours or extreme lethargy will be considered as humane endpoints. If it is decided to euthanize the animal, it will be euthanized under anesthesia and the transplanted graft will be harvested at that time point. Euthanasia was performed by cervical dislocation under deep anesthesia induced by isoflurane or CO2. We performed 12 transplantations using 24 mice and used 6 mice for sham group. Only one mouse after transplantation was euthanized because of respiratory difficulty at 2POD (It was excluded in this study). No mice died before meeting criteria for euthanasia.

### Pathological evaluation

Lung grafts were harvested on day 21 post-surgery, fixed in glutaraldehyde, and paraffin embedded. Each lung was sectioned and stained with hematoxylin/eosin and Masson Trichrome stain to evaluate the presence of airway fibrosis and to diagnose OB. The analysis was performed by two independent investigators (AH and SH) in conjunction with an experienced pathologist (MK), as described previously [19].

### RNA extraction

Only animals diagnosed histologically were subjected to the gene expression analysis for each group. All samples were immediately frozen at -80°C with Allprotect Tissue Reagent (Qiagen, Hilden, Germany) according to the manufacturer’s protocol. Total RNA from each sample was isolated by homogenization of the frozen tissue and purified.

### Comprehensive microarray analysis

Independently purified mRNA from individual representative mice of OB(n = 3), non-OB(n = 3), and sham mice(n = 3) was subjected to microarray analysis using an Agilent SurePrint G3 Mouse Gene Expression 8X 60K array (Agilent Technologies). The scanned images were analyzed with Feature Extraction Software 12.0.3.1(Agilent) using the default parameters to obtain background subtracted and spatially detrended processed signal intensities. The processed signal intensities were then normalized using the global scaling method. The microarray data have been lodged in the Gene Expression Omnibus (http://www.ncbi.nlm.nih.gov/geo/) as accession number: GSE137169.

### Hierarchical clustering analysis

Hierarchical clustering was carried out based on the city-block distance and complete linkage clustering algorithms in both sample and probe directions using Cluster 3.0 software [20]. The heat map was drawn using Java Tree View software [21].

### Identification of upregulated and downregulated transcripts in OB

To identify regulated transcripts in the OB samples, gene expression was compared to the sham group and the fold change for each gene was calculated. Student’s *t* tests were performed on normalized data processed with log2 transformation of the normalized signal intensities. The thresholds used to determine differential transcript expression were a mean fold change ≥ 2-fold or ≤0.5-fold and a *P* value <0.05 (S1 Fig). This analysis identified 2,169 upregulated transcripts in the OB samples compared to the sham. To compare the OB samples to the non-OB samples, these 2,164 upregulated transcripts were again analyzed in terms of their fold change and Student’s *t*-tests (*P* < 0.05). Gene ontology (GO) term in biological processes (GOTERM_BP_FAT) enrichment was then analyzed for the extracted gene lists using the Functional Annotation tool at DAVID Bioinformatics Resources (http://david.abcc.ncifcrf.gov/). A significant change in the GO analysis was set at *P* < 1.0 × 10^−5^.

### Quantitative real-time PCR

For validation with additional samples (n = 5–6 each), we verified the expression of toll like receptor 2 (*Tlr2*), C-C motif chemokine ligand 3 (*Ccl3*), histocompatibility 2, class II antigen A, beta 1 (*H2-ab1*), interleukin-21 (*Il-21*), immunoglobulin heavy constant gamma 3 (*Ighg3*), interferon gamma (*Ifng*), programmed cell death 1 (*Pdcd1*), and programmed cell death 1 ligand 1 (*Pdcd1lg1*) in the LN, spleen, and lung samples. These genes were related to significantly enriched GO term in OB or representative genes of cellular or humoral rejection. The cDNA was then amplified using primers purchased from Qiagen (listed in S1 Table) in conjunction with a QuantiTect SYBR Green PCR kit (Qiagen). Quantification of gene expression was performed with a sequence-detection system (CFX96 Touch^™^ Real-Time PCR Detection System; Bio-Rad Laboratories, Hercules, CA) according to the manufacturer’s protocol. Target gene mRNA expression was calculated with calibration curves for each individual gene using standard samples and was normalized to that of the housekeeping gene beta-actin (*Actb*). The experiment was duplicated, and the mean and standard error were calculated.

### Immunohistochemical staining

Tissue staining was then performed with the following primary antibodies per the manufacturer’s protocols: rat monoclonal anti-H2-Ab1 antibody (#77–190; Prosci Inc, Poway, USA), rabbit monoclonal anti-CD3 antibody (#ab16669; Abcam plc, Cambridge, UK), rat monoclonal anti-CD45R/B220 antibody (#MCA1258G; Bio-Rad Laboratories).

### Statistical analysis

Differences in gene expression by qPCR were tested individually using the Steel test. The expression in the sham group was used as a control. Statistical analysis was performed using JMP (version 15.0). *P* < 0.05 was considered statistically significant. Hierarchical clustering was performed using Cluster 3.0 software [20]. The heat maps were drawn with Java Tree View software [21].

## Results

### Evaluation of the murine orthotopic lung transplantation model

**Fig 2:**
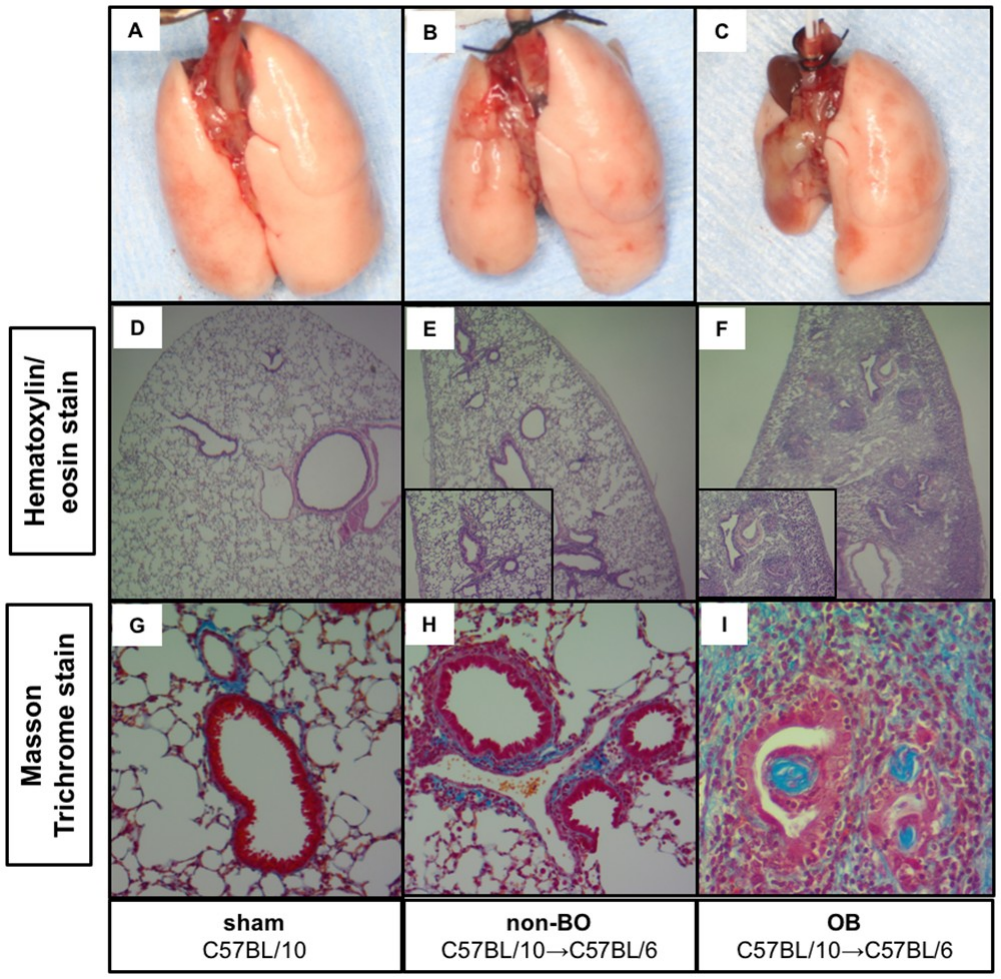
Histological feature of the lung grafts for each experimental group. Representative macroscopy (A-C), hematoxylin/eosin stained tissue (D-F), and Masson trichrome stained (G-I) images for the sham (A, D, G), non-OB (B, E, H), and OB (C, F, I) groups. Black arrow indicates transplanted left lungs in OB and non-OB. Compared to the sham group, the non-OB lung grafts had relatively normal morphology with a slight increase in the number of inflammatory cells, while the OB lung grafts had severe fibrosis, infiltrating inflammatory cells, and fibrotic polyps in the bronchi.

The histology of the left lungs in the sham, non-OB, and OB groups are shown in Fig 2. None of the morphological changes in the OB and non-OB groups were observed in the sham-treated mice (Fig 2A, 2D and 2G). Fibrotic polyps were observed in the bronchi of the mice with OB following orthotopic lung transplantation (Fig 2F and 2I). Polyp formation was also accompanied by an increased number of infiltrating lymphocytes and macrophages. Alternatively, in the non-OB mice, normal lung morphology was maintained, although an increased number of mononuclear cells was observed around the bronchi (Fig 2B, 2E and 2H).

**Fig 3:**
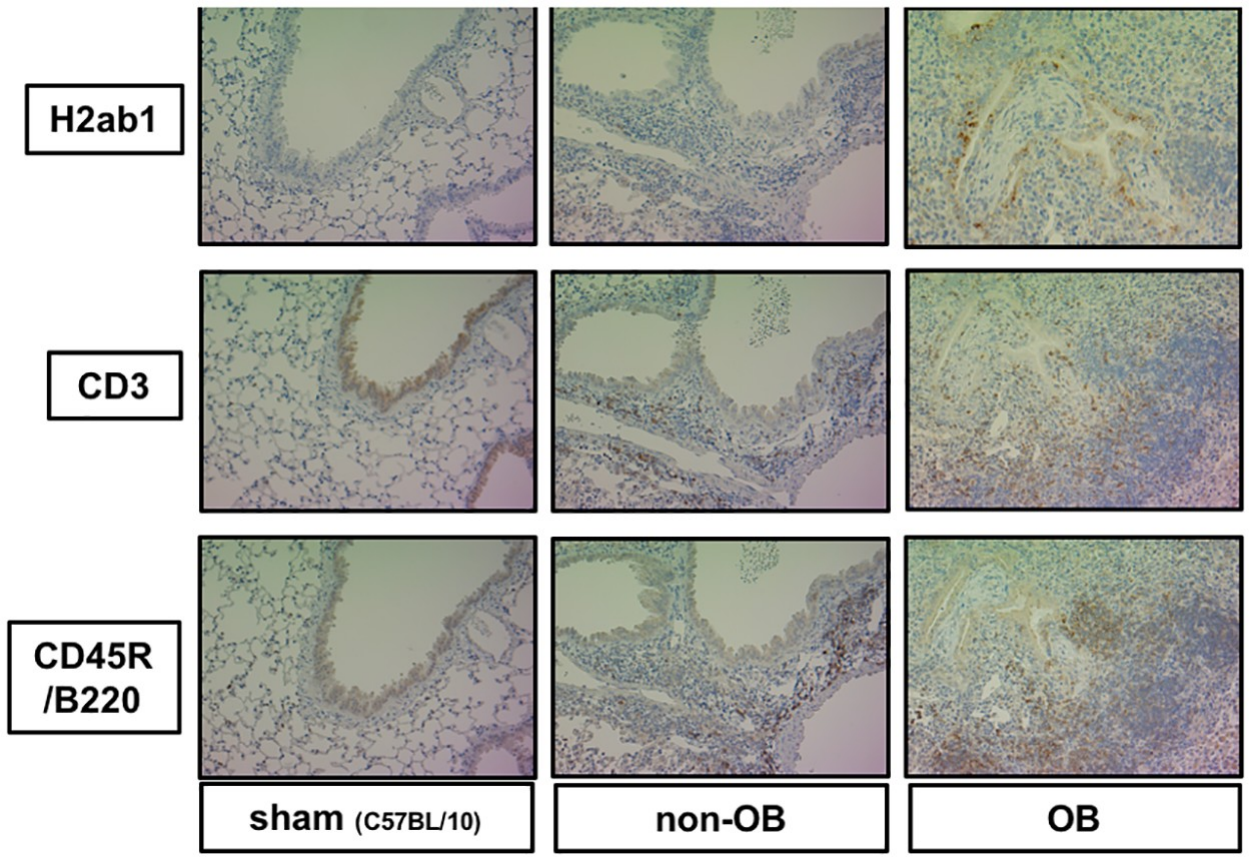
Representative immunohistochemical photomicrographs from allografts and lungs for the three experimental groups. (A-C) H2-ab1, an MHC class II molecule, is intensely expressed in the bronchial epithelium in the bronchi of OB mice. Infiltrating lymphocytes were stained with CD3 (D-F), the common receptor for T cells, or CD45R/B220 (G-I), the common receptor for B cells (J-L).

Bronchial epithelial cells and mononuclear cells, such as macrophages, had intense positive staining for H2-Ab1 in the transplant mice with OB (Fig 3). Indeed, many infiltrating lymphocytes were observed around the bronchi that had fibrotic polyps, and they were positive for CD3 or CD45R/B220, which suggests the presence of B cells and T cells. In the non-OB transplant mice, H2-Ab1 staining in the bronchial epithelium was also observed as well as CD3, CD45R/B220 in the inflammatory cells. However, this staining was qualitatively less intense compared to that in the OB lungs. No positive staining was observed for these antibodies in the sham group.

### Differential gene expression in lungs with OB after transplantation

To get a broad understanding of gene expression, an unsupervised two-way hierarchical clustering analysis was performed on the whole lung grafts (S2 Fig). As the lungs from both inbred mouse strains showed similar profiles after the sham surgery, both types were used as controls.

**Fig 4:**
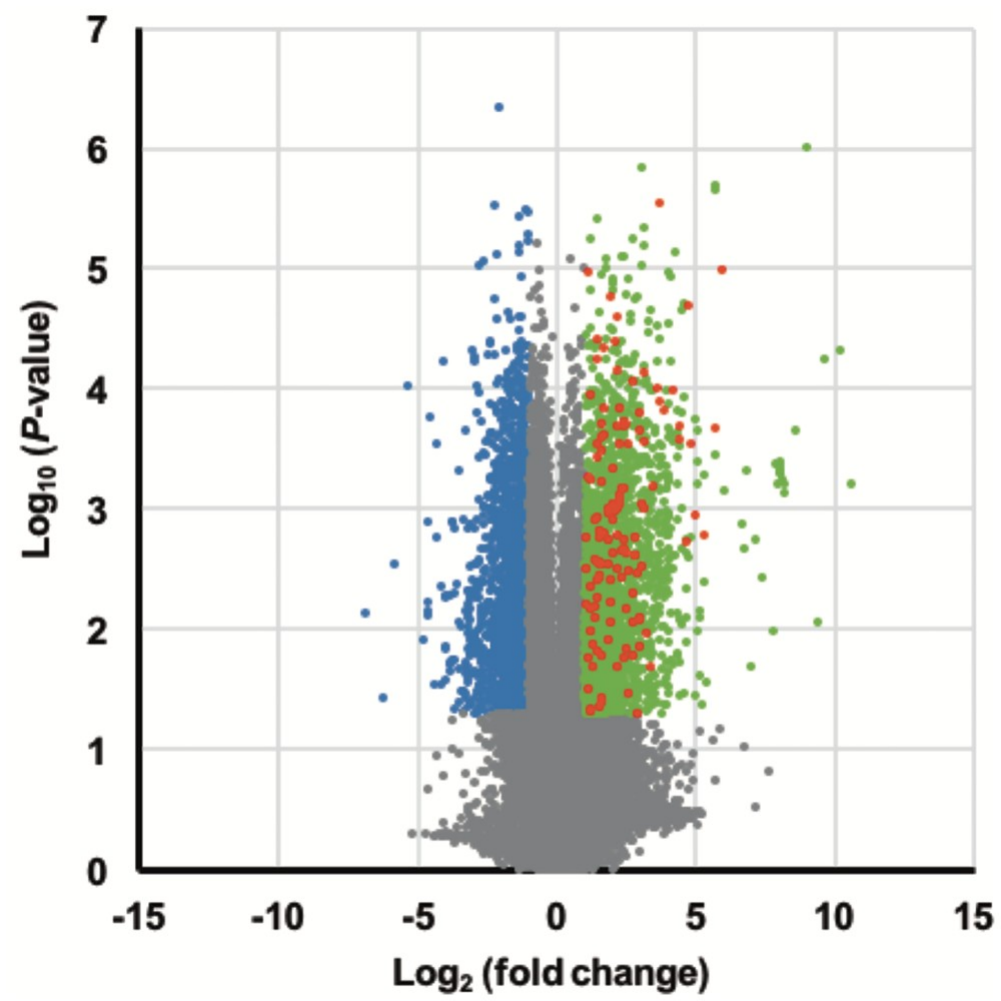
Volcano plot of differential gene expression in the OB lung grafts. The differences in the mean signal intensities are shown using several colors: blue, downregulated compared to the expression in sham surgery grafts; green, upregulated compared to the expression in sham surgery grafts; red, upregulated compared to the expression in non-OB lung grafts.

**Fig 5:**
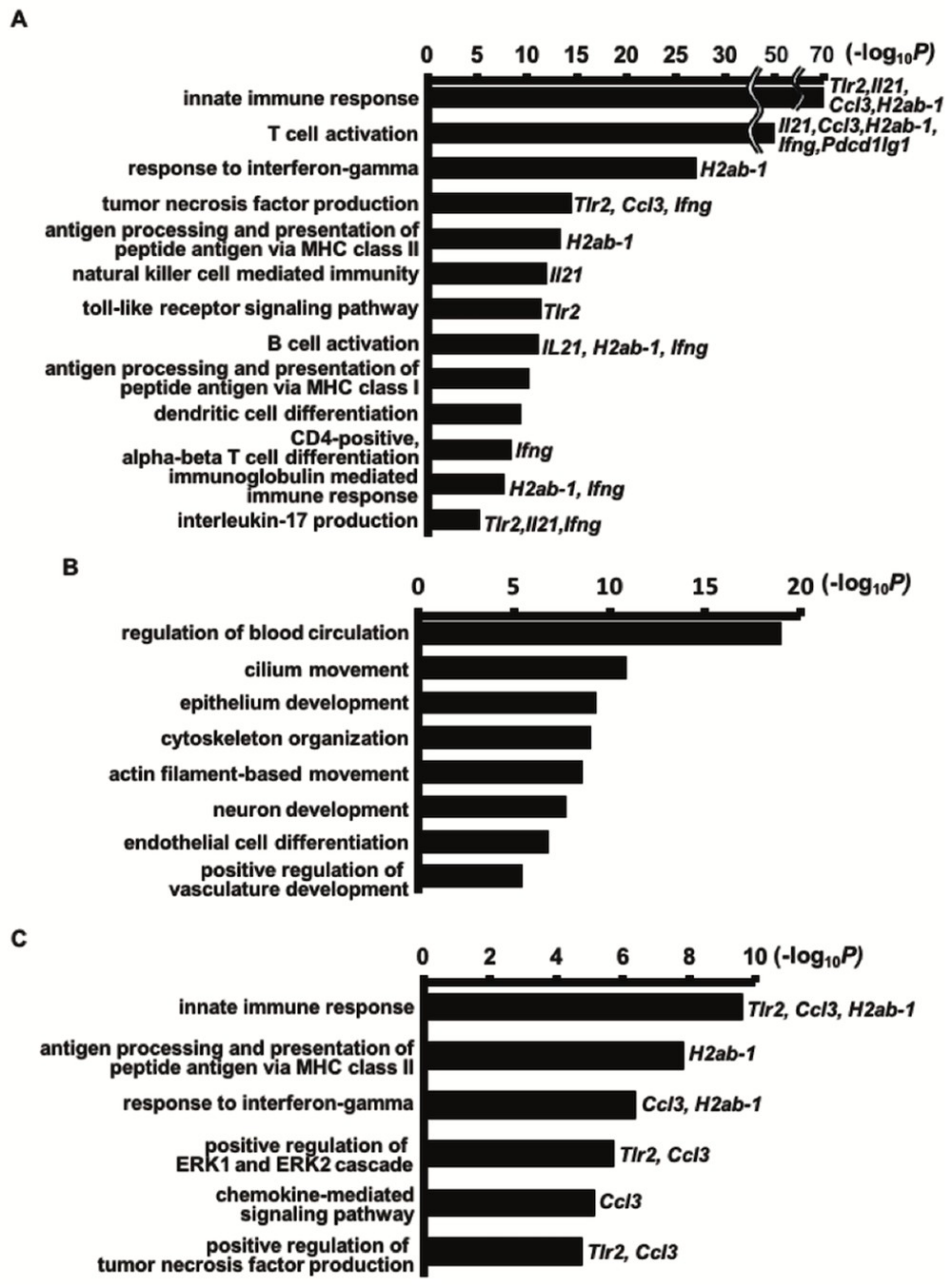
Functional analysis of the regulated genes in the OB lung grafts. Lung grafts were harvested at day 21 after lung transplantation, and their regulated genes were identified by microarray analysis. A mean fold change greater than 2.0 or less than 0.5 compared to the sham was required. Significance was set at *P* < 0.05. Upregulated (A) and downregulated (B) genes are shown compared to the sham group, as well as the upregulated genes compared to the non-OB group (*P* < 0.05) (C). Representative upregulated genes are indicated next to each gene ontology (GO) term bar.

A volcano plot is shown in Fig 4, giving an overview of the regulated genes in the OB group. Compared to the sham group, there were 2,169 transcripts (1,343 genes) upregulated in the OB mice (S2 Table). These genes appear to be highly related to the innate (e.g. *Tlr2* and *CCL3*) and adaptive immune responses (e.g. *H2Ab-1* and *Il-21*), as highlighted by our GO term enrichment analysis (Fig 5A). Notably, the innate immune response includes natural killer (NK) cell-mediated immunity and the toll-like receptor (TLR) signaling pathway, both of which involve genes that were upregulated in the OB tissues. In contrast, the adaptive immune response appears to be mainly related to genes involved in the presentation of MHC class II molecules during B cells and T cell activation as well as those involved in the immunoglobulin-mediated immune response.

Furthermore, compared to the sham group, 2,225 transcripts (1,587 genes) were downregulated in the OB mice (S3 Table). These genes appear to be largely related to cilium movement, epithelium development, and endothelial cell differentiation (Fig 5B). It is also important to note that 127 transcripts (95 genes) were upregulated in the OB group compared to the non-OB group (S4 Table), with the majority of the genes being related to innate immunity as well as the processes of antigen processing and presentation of MHC class II molecules (Fig 5C). These data indicate that while the non-OB group may have some altered gene expression following lung transplantation, the immune response is not as activated as that in the OB group.

### Upregulation was verified for select genes in the lung grafts, LNs, and spleens using qPCR

**Fig 6:**
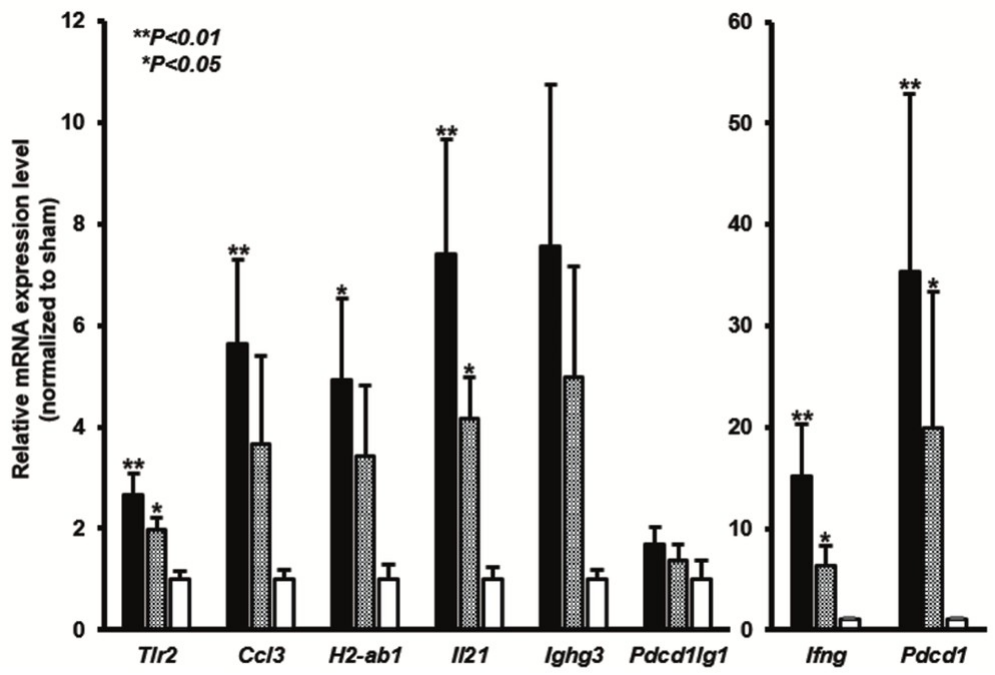
The mRNA expression of representative genes was verified in lung grafts from OB mice. The microarray data is shown (A). The expression of *Tlr2*, *Ccl3*, *H2-ab1*, *Il21*, *Ighg3*, *Ifng*, *Pdcd1*, and *Pd1lg1* were examined by qPCR in the sham, non-OB, and OB groups (B). The mean mRNA expression of each gene in the sham group was defined as 1 and all the other expression values were calculated relative to this level. Bars represent the means ± SE from at least 5 individual mice in each group. Black bar, OB; grey bar, non-OB; white bar, sham. **P < 0.01, *P < 0.05, compared to the sham group.

**Fig 7:**
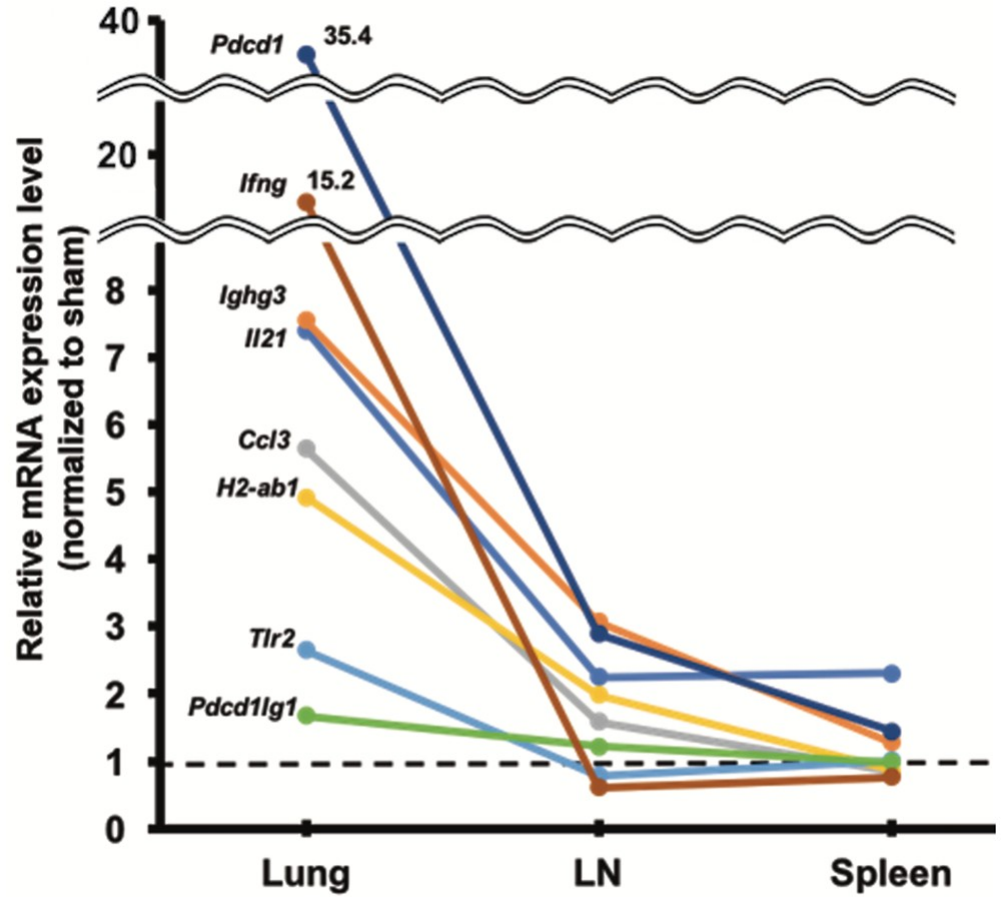
The mRNA expression of representative genes in lung grafts, lymph nodes, and spleens of OB mice compared to that of sham mice. The mean mRNA expression of each gene in the sham group was defined as 1 and all the other expression values were calculated relative to this level. The differences in gene expression between the OB and sham groups were reduced in the left mediastinal lymph nodes and spleens compared to the differences in lung graft expression. However, some genes, such as *Pdcd1*, *Ighg3*, and *Il21*, were still upregulated above 2-fold in the left mediastinal lymph nodes.

**Fig 8:**
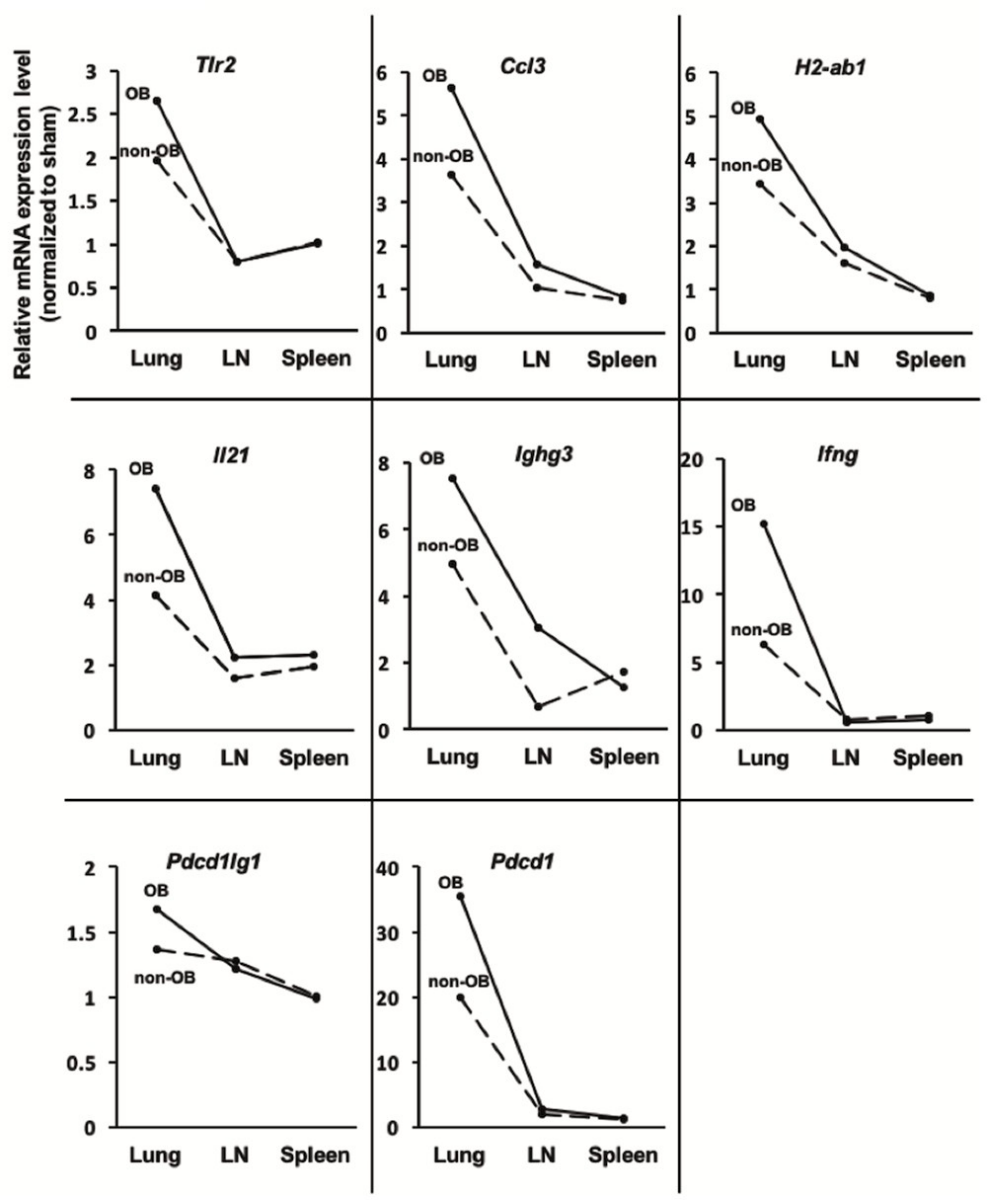
The mRNA expression of representative genes in lung grafts, lymph nodes, and spleens of OB mice compared to that of non-OB mice. The mean expression of each gene in the sham group was defined as 1 and all the other expression values were calculated relative to this level. The expression of *Ighg3*, *Il-21*, *Ccl3*, *H2-ab1*, and *Pdcd1* was more upregulated in the mediastinal lymph nodes as well as the lungs of OB mice compared to that observed in non-OB mice.

In the lung grafts, *Tlr2*, *Ccl3*, *H2Ab1*, *Il21*, *Ifng*, and *Pdcd1* expression was significantly upregulated in the OB group compared to the sham group (*P* < 0.05) (Fig 6). Further, *Tlr2*, *Il21*, *Ifng*, and *Pdcd1* expression in the non-OB lung grafts was also significantly upregulated compared to that in the sham group, although the levels were still relatively low compared to the OB group. In the LNs and spleens, expression of all of these target genes was reduced in the OB group; however, that of *Pdcd1*, *Ighg3*, and *Il21* were still 2.0-fold overexpressed in the LNs, and *Il21* was still 2.0-fold overexpressed in the spleens (Fig 7). Compared to the non-OB group, the expression of *Ighg3*, *Il21*, *Ccl3*, and *H2-ab1* was upregulated in both the lung grafts and LNs of the OB group, while the expression of all of the genes was similar in the spleen (Fig 8).

## Discussion

OB is a major characteristic of BOS [10], which is the most significant barrier to long-term lung transplant patient survival [1, 2]. Microarray analysis was recently applied to evaluate OB-mediated gene expression changes [13–16]. As animal models are a useful tool for studying OB, we utilized microarray analysis to study gene expression in our recently developed murine OB orthotopic lung transplantation model. We observed significant histological differences in the OB lung grafts compared to the sham group, while less severe differences were observed for the non-OB grafts. This was also reflected in gene expression. This is the first study evaluating comprehensive gene expression during OB caused by mHA disparity in a murine orthotopic lung transplantation model.

mHA is defined as a non-MHC antigenic gene product that induces CD4^+^ and CD8^+^ T cell infiltration, triggering an immune response [22]. mHA is also presented by MHC class II antigen-presenting cells (APCs) of the donor or recipient [23]. Furthermore, mHA disparity has been shown to induce cellular and humoral immune rejection of kidney transplantation [23–25]. For example, non-human leukocyte antigen (non-HLA) antibodies are an important factor in renal transplants [24, 26] and the number of mismatched HLAs is associated with survival, with no mismatches having the best chance for survival [27]. Surprisingly, recent studies have shown that non-MHC mismatched cases also have a high risk of developing BOS [28, 29], indicating this pathology as a factor in the poor outcome of many lung transplants. These data also suggest that an immune response other than MHC disparity, such as non-HLA antibody-related immune changes, is crucial for BOS. Therefore, the present study using microarray analysis to evaluate broad gene expression changes in our recently developed OB model was performed to elucidate the immunological mechanisms of BOS pathogenesis.

Intensive staining of H2-ab1 was observed in our OB mice, similar to previous studies [30, 31]. Various mediators of inflammation, such as ischemia reperfusion injury (IRI) and viral infection, can upregulate MHC molecules [32, 33]. For example, interferon-γ induces *de novo* MHC class II antigen expression in non-APCs [34, 35], and a significant upregulation of *Ifng* expression was observed after treatment. Notably, H2-ab1 over-expression in the bronchial epithelium may not be crucial for OB development in our model because it is an MHC-matched model. However, some studies have shown that the amount of MHC antigen was related to the severity of the bronchial injury [32, 36], and that MHC class II mismatch and anti-MHC class II antibodies in the donor play a more important role than MHC class I in human lung [37, 38] and kidney transplants [39, 40]. Furthermore, MHC class II over expression in bronchial epithelium might impaired the inhibitory effect to T cell proliferation in the bronchial epithelium cells [41]. Therefore, immune mediators, such as interferon γ, could induce the expression of MHC class II antigens regardless of MHC disparity and inhibiting these factors could be an efficient treatment for chronic transplant rejection, especially AMR.

B cell infiltration as well as *Il21* and *Ighg3* upregulation was also observed in the OB mice. IL21 is a pro-inflammatory cytokine that is strongly related to transplant immunology [42]. It is also an important mediator of follicular T cell function and promotes B cell differentiation and immunoglobulin production [43]. *Ighg3* encodes IgG3, which is a member of one of the most effective subclasses of humoral immunity and is involved in both complement activation and antibody-dependent cellular cytotoxicity (ADCC) during transplantation [44, 45]. Changes in these genes highlight distinct changes in immune function that greatly affect transplant effectiveness.

Notably, the innate immune response was the predominant pathway highlighted by the upregulated genes in the OB mice. *Ccl3* and *Tlr2* were verified as representative genes of this pathways. *CCL3* has been shown to be upregulated in ADCC during renal transplantation [46], but the present study is the first to demonstrate a change it its expression during BOS. ADCC is initiated by the Fc portion of antibodies interacting with leukocytes, such as NK cells, via Fc-receptors [47], and it plays an important role in AMR [48]. The role of an NK cell-mediated pathway in OB is supported by a recent report [49]. Moreover, TLRs also play a central role in the innate immune response, especially for IRI [50]. Interestingly, TLR2 is upregulated in IRI and is related to BOS development [51, 52]. An antagonist of TLR2 was also clinically applied to enhance outcome for renal transplant patients [53]. These data indicate a significant role for the innate immune response during OB/BOS development.

Further, in addition to the innate immune response, this study also revealed a role for adaptive immunity during OB. Indeed, a number of representative genes were upregulated during OB, although they were also significantly upregulated in non-OB mice, compared with their expression in the sham controls. Youssef et al. [54] similarly observed that the degree of antigenic disparity between donor and recipient, fully allogenic compared with mHA-mismatched, did not influence the expression of all cellular immune responses during graft rejection following skin and heart transplant. Although they evaluated gene expression by semi-quantitative PCR, our quantitative microarray and qPCR analyses provide more detail concerning the moderate immune response in non-OB transplants. Our results suggest that bronchial epithelium lysis leading to OB development depends on the degree of both innate and adaptive immunity, and the response level may be regulated by non-immune reactions to the graft, such as reperfusion or infection.

The representative gene verified to evaluate adaptive immunity was *Pdcd1*. The pathway involving PD-1, PD-L1, and PD-L2 has been identified as a potential biomarker of immunotolerance during transplantation [55, 56]. In fact, PD-1 is overexpressed during T cell exhaustion, which is a state of T cell dysfunction characterized by progressive loss of function following chronic antigenic stimulation. Elevated urinary PD-1 mRNA levels have also been associated with acute rejection [55]. Moreover, PD-1 expression on CD8+ T cells is critical for tolerance induction *in vivo* [56]. These data as well as ours demonstrate that PD-1 expression in lung grafts could be used as a potential biomarker of OB.

It is important to note that *Il-21*, *Ighg3*, and *Pdcd1* were upregulated in the LNs as well as the lung grafts of OB mice. The LNs are considered to be an important site of antigen presentation and lymphocyte maturation. This is particularly important during OB diagnosis as its heterogeneous distribution makes detection with traditional TBB difficult. Thus, LN biopsy using endobronchial ultrasound-guided transbronchial needle aspiration could circumvent these issues. Additional work is required to evaluate LN biopsy as a potential diagnostic tool for OB.

The current study does have some limitations. First, the number of animals in this study was relatively low, which caused statistical limitation. Three animals were only used for microarray analysis, so we couldn’t evaluate with standard threshold such as false discovery late. However, to overcome this limitation, five or six animals were used for validation in qPCR and we validated the expression level in local and systemic lymphoid tissue as well as lung. Some genes were upregulated in LNs of OB, which is not previously described. To reduce the number of animals in terms of animal protection, the further investigation is needed using clinical lung samples. Second, this study analyzed whole lungs, which contain various cell types, such as fibroblasts, endothelial cells, and immune cell. Further investigation of the infiltrating cells should be needed by flow cytometry. Third, we did not evaluate the cellular reaction between the donor and recipient *in vitro*. Some reports indicate that the origin of the infiltrating fibroblasts and lymphocytes into the OB lesion is important [57, 58]. We speculate that the lung grafts contain both donor and recipient cells. Therefore, we used the lungs from both C57BL/6 and C57BL/10 as a control after confirming their similar gene expression after sham surgery. Finally, the entire analysis was performed only at day 21 after transplantation. That may explain why known rejection signals, such as TGF-β signaling, which occur earlier were not observed in this study. Fibrosis gene expression also possibly occurs prior to histological changes [59] and may have also been missed. Thus, other time points should be investigated to evaluate earlier gene expression changes as well as the duration of gene upregulation of the immune-related genes found in this study. Further study is needed with physiological evaluation (e.g. spirometry) as well as molecular analysis to develop the early detection tool for clinical use.

## Conclusions

In this study, we performed the first comprehensive gene expression analysis in OB after murine orthotopic lung transplantation. Our results suggest that mHA disparity induced adaptive and innate immune responses caused by humoral rejection as well as cellular rejection. Moreover, some novel genes related to these pathways were upregulated in the LNs as well as the lung grafts of OB mice. Taken together, our findings provide insight into the mechanism underlying OB development and can be used to help develop diagnostic tools and treatments for BOS.

## Supporting information

S1 Data Supplemental material and methods. (PDF)

S2 Data *PLOS ONE* humane endpoints checklist. (DOCX)

S1 Fig Marker extraction of BO. To identify regulated transcripts in the data processing, the fold changes between the compared groups and *P* values with *t* test were calculated. The *t* test was performed on normalized data processed with log2 transforming. The threshold for selecting regulated transcripts was set at a ≥ 2-fold or ≤0.5-fold change and a *P* value <0.05 from sham group at mean value. For compared to non-OB, upregulated transcripts were extracted with *P*<0.05 among those 2,169 upregulated transcripts.
(PDF)

S2 Fig Unsupervised two-way hierarchical clustering of all six samples. Unsupervised two-way hierarchical clustering based on standard deviation (above 1.8) classified lung samples into two groups: allografts and sham group. The two types of sham, C57BL/10 and C57BL/6, had similar mRNA expression compared to allografts.
(PDF)

S1 Table Primers for qPCR. (XLSX)

S2 Table Gene list showing at least two-fold upregulation in OB group compared to sham group. (XLSX)

S3 Table Gene list showing at least two-fold down regulation in OB group compared to sham group. (XLSX)

S4 Table Gene list showing upregulation in OB group compared to non-OB group. (XLSX)
