# Supplementary material for: Differential gene analysis during the development of obliterative bronchiolitis in a murine orthotopic lung transplantation model: A comprehensive transcriptome-based analysis
Source: PLoS One. 2020 May 8;15(5):e0232884. doi: 10.1371/journal.pone.0232884 (PMC7209239; doi:10.1371/journal.pone.0232884)
Supplement: S1 Data — (PDF) [file pone.0232884.s001.pdf]

Supplemental Information

Differential gene analysis during the development of obliterative bronchiolitis in a murine orthotopic lung transplantation model: a comprehensive transcriptome-based analysis

Author: Atsushi Hata[1], Hidemi Suzuki[1], Takahiro Nakajima[1], Taiki Fujiwara[1], Yuki Shiina[1], Taisuke Kaiho[1], Takahide Toyoda[1.2], Terunaga Inage[1], Takamasa Ito[1], Yuichi Sakairi[1], Hajime Tamura[1], Hironobu Wada[1], Yoshito Yamada[1], Masako Chiyo[1], Keisuke Matsusaka[3], Masaki Fukuyo[3,4], Ken-ichi Shinohara[3], Sakae Itoga[5], Shinichiro Motohashi[2], Kazuyuki Matsushita[5], Atsushi Kaneda[3], Ichiro Yoshino[1]

[1]Department of General Thoracic Surgery, Chiba University Graduate School of Medicine, Chiba, Japan

[2]Department of Medical Immunology, Chiba University Graduate School of Medicine, Chiba, Japan

[3]Department of Molecular Oncology, Chiba University Graduate School of Medicine, Chiba, Japan

[4]Department of Genome Research and Development, Kazusa DNA Research Institute

[5]Department of Laboratory Medicine & Division of Clinical Genetics and Proteomics, Chiba University Graduate School of Medicine, Chiba, Japan

Correspondence information: Hidemi Suzuki, MD, PhD,

Department of General Thoracic Surgery, Graduate School of Medicine, Chiba University

1-8-1 Inohana, Chuo-ku, Chiba, 260-8670 Japan

Tel +81-43-222-7171 (ext. 5464); Fax +81-43-226-2172

E-mail: hidemisuzukidesu@yahoo.co.jp

Supplemental Materials and Methods

Animals

Specific pathogen-free male inbred C57BL/6 ($H2^b$) and C57BL/10($H2^b$) mice were purchased from CLEA Japan, Inc. (Tokyo, Japan) and the Central Institute for Experimental Animals (Kanagawa, Japan), respectively. They were housed at the Biomedical Research Center at Chiba University School of Medicine in accordance with institutional guidelines. C57BL/10 ($H2^b$) were used as donors and C57BL/6($H2^b$) as recipients at 8–12 weeks of age (body weight, 24–32 g).

Surgical technique

The orthotopic transplantation of the left lung was performed as described previously.[1] Briefly, the left lung of donor was harvested and prepared for the recipient by the attachment of cuffs made from intravenous catheter (pulmonary artery (PA), 20-guage; pulmonary vein (PV), 22-gauge; bronchus (Br), 20G, respectively). After left thoracotomy and gentle traction of hilar PA, PV and Br were performed for the recipient, the donor lung was transplanted by inserting cuffs into the recipient PA, PV and Br. All surgical procedures were performed by A.H. utilizing sterile techniques. Only thoracotomy was performed for sham. Buprenorphine (0.05–

0.15 mg/kg) was administered immediately after surgery and every 8 hours for 2–3 days post-surgery. The transplanted lung grafts and the left lungs for sham group were harvested 21 days post-surgery along with the mediastinal lymph nodes (LNs) and spleens. Each lung was separated into two specimens for pathological examination and gene profiling.

Pathological evaluation

Lung grafts were harvested, fixed in glutaraldehyde, and paraffin embedded. Each lung was sectioned and stained with hematoxylin/eosin and Masson Trichrome stain to evaluate the presence of airway fibrosis and to diagnose OB. The analysis was performed by two independent investigators (AH and SH) in conjunction with an experienced pathologist (MK), as described previously.[2]

RNA extraction

All samples were immediately frozen at -80°C with Allprotect Tissue Reagent (Qiagen, Hilden, Germany) according to the manufacturer's protocol. For microarray analysis, total RNA from the samples was isolated by homogenization of the frozen tissue using TissueLyser II (Qiagen) and purified by NucleoSpin® RNA XS (MACHEREY-NAGEL) as described in the

manufacturer’s protocol. For quantitative real-time PCR (qPCR), total RNA from each lung graft, LN, and the spleen was isolated and purified using a MagNA Lyser® and MagNA Pure Compact RNA Isolation Kit (Roche Diagnostics PLEASANTON, United States) per the manufacturer’s protocol.

Comprehensive microarray analysis

Purified mRNA from nine lung grafts (OB, non-OB, and sham; n = 3 in each group) were subjected to a microarray analysis with a coverage of 59,305 transcripts (Agilent SurePrint G3 Mouse Gene Expression 8× 60K arrays; Agilent Technologies) as follows.

Cyanine-3 (Cy3) labeled cRNA was prepared from 0.1 μg of total RNA using a Low Input Quick Amp Labeling Kit (Agilent) according to the manufacturer's instructions. These labeled cRNAs were then purified with an RNeasy column (Qiagen, Valencia, CA). Dye incorporation and cRNA yield were checked with a NanoDrop ND-2000 Spectrophotometer. A total of 0.6 μg of Cy3-labeled cRNA was then fragmented at 60°C for 30 minutes in a reaction volume of 25 μl containing 1× Agilent fragmentation buffer and 2× Agilent blocking agent following the manufacturer’s instructions. After fragmentation, 25 μl of 2× Agilent hybridization buffer was added to the fragmentation mixture and hybridized to SurePrint G3 Mouse Gene Expression

8× 60K arrays (Agilent Technologies) for 17 hours at 65°C in a rotating Agilent hybridization oven. After hybridization, microarrays were washed for 1 minute at room temperature with GE Wash Buffer 1 (Agilent), followed by a 1 minute wash at 37°C with GE Wash buffer 2 (Agilent). Immediately after washing, the slides were scanned on an Agilent SureScan Microarray Scanner (G2600D) using the one color scan setting for 8× 60k array slides (Scan Area, 61 × 21.6 mm; Scan resolution, 3 μm; Dye channel, Green Photomultiplier tube is set to 100%).

The scanned images were analyzed with Feature Extraction Software 12.0.3.1(Agilent) using the default parameters to obtain background subtracted and spatially detrended processed signal intensities. The processed signal intensities were then normalized using the global scaling method. A trimmed mean probe intensity was determined by removing 2% of the lower and higher ends of the probe intensities and was then used to calculate the scaling factor. Normalized signal intensities were then calculated from the target intensity on each array using the scaling factor, whereby the trimmed mean target intensity of each array was arbitrarily set to 2500.

Hierarchical clustering analysis

Hierarchical clustering was carried out based on the city-block distance and complete linkage

clustering algorithms in both sample and probe directions using Cluster 3.0 software.[3] The heat map was drawn using Java Tree View software.[4]

Identification of upregulated and downregulated transcripts

To identify regulated transcripts in the OB samples, gene expression was compared to the sham group and the fold change for each gene was calculated. Student's *t* tests were performed on normalized data processed with log2 transformation of the normalized signal intensities described above. The thresholds used to determine differential transcript expression were a mean fold change ≥ 2-fold or ≤0.5-fold and a P value <0.05. This analysis identified 2,164 upregulated transcripts in the OB samples compared to the sham. To compare the OB samples to the non-OB samples, these 2,164 upregulated transcripts were again analyzed in terms of their fold change and Student's *t*-tests ($P < 0.05$). Gene ontology (GO) term in biological processes (GOTERM_BP_FAT) enrichment was then analyzed for the extracted gene lists using the Functional Annotation tool at DAVID Bioinformatics Resources (http://david.abcc.ncifcrf.gov/). A significant change in the GO analysis was set at $P < 1.0 \times 10^{-5}$.

Quantitative real-time PCR (qPCR)

For validation with additional samples (n = 5–6 each), we verified the expression of toll like receptor 2 (*Tlr2*), C-C motif chemokine ligand 3 (*Ccl3*), histocompatibility 2, class II antigen A, beta 1 (*H2-ab1*), interleukin-21 (*Il-21*), immunoglobulin heavy constant gamma 3 (*Ighg3*), interferon gamma (*Ifng*), programmed cell death 1 (*Pdcd1*), and programmed cell death 1 ligand 1 (*Pdcd1lg1*) in the LN, spleen, and lung samples. For qPCR, 500 µg of total RNA (100 µg for LN) was treated with RNase-free DNase I to remove genomic DNA and was reverse-transcribed using a QuantiTect Reverse Transcription kit (Qiagen). Notably, for *Il-21* and *Ifng*, 1000 µg of total RNA (100 µg for LN) was used for reverse-transcription because these genes are expressed at relatively low levels in all of the tissues examined. The cDNA was then amplified using primers purchased from Qiagen (listed in Supplementary Table S1) in conjunction with a QuantiTect SYBR Green PCR kit (Qiagen). Quantification of gene expression was performed with a sequence-detection system (CFX96 Touch™ Real-Time PCR Detection System; Bio-Rad Laboratories, Hercules, CA) according to the manufacturer's protocol. Target gene mRNA expression was calculated with calibration curves for each individual gene using standard samples and was normalized to that of the housekeeping gene beta-actin (*Actb*). If the expression level of the sample was under the lower limit of

quantification, it was expressed as the minim expression level of standard used in each calibration curve. The relative expression was scaled such that the mean expression of each gene in the sham group equaled one. The experiment was duplicated, and the mean and standard error were calculated.

Immunohistochemical staining

For immunohistochemical staining, paraffin sections were cut (about 3 mm in thickness) and mounted on silane-coated slides. Tissue staining was then performed with the following primary antibodies per the manufacturer's protocols: rat monoclonal anti-H2-Ab1 antibody (#77-190; Prosci Inc, Poway, USA), rabbit monoclonal anti-CD3 antibody (#ab16669; Abcam plc, Cambridge, UK), rat monoclonal anti-CD45R antibody (#MCA1258G; Bio-Rad Laboratories), and rabbit polyclonal anti-C4d antibody (#HP8033; Hycult biotech, Uden, NL). The immunoreaction was visualized using peroxidase-diaminobenzidine (DAB). The sections were counterstained with hematoxylin.

Statistical analysis

Differences in gene expression by qPCR were tested individually using the Steel test. The

expression in the sham group was used as a control. Statistical analysis was performed using JMP (version 15.0). $P < 0.05$ was considered statistically significant. Hierarchical clustering was performed using Cluster 3.0 software [3]. The heat maps were drawn with Java Tree View software [4].

Supplemental References

1. Suzuki H, Fan L, Wilkes DS: Development of obliterative bronchiolitis in a murine model of orthotopic lung transplantation. J Vis Exp 2012.

2. Fan L, Benson HL, Vittal R, et al.: Neutralizing IL-17 prevents obliterative bronchiolitis in murine orthotopic lung transplantation. Am J Transplant 2011;11:911-22.

3. de Hoon MJ, Imoto S, Nolan J, Miyano S: Open source clustering software. Bioinformatics 2004;20:1453-4.

4. Saldanha AJ: Java Treeview--extensible visualization of microarray data. Bioinformatics 2004;20:3246-8.
