# Supplementary material for: Differential gene analysis during the development of obliterative bronchiolitis in a murine orthotopic lung transplantation model: A comprehensive transcriptome-based analysis
Source: PLoS One. 2020 May 8;15(5):e0232884. doi: 10.1371/journal.pone.0232884 (PMC7209239; doi:10.1371/journal.pone.0232884)
Supplement: S2 Data — (DOCX) [file pone.0232884.s002.docx]

*PLOS ONE* Humane Endpoints Checklist

*PLOS ONE* manuscript number: PONE-D-19-24392

**Complete the following if your study design includes death of a regulated animal as a likely outcome or planned experimental endpoint. Please also include all information in the Methods section of your manuscript.**

**ITEM 1.** **Describe whether humane endpoints* were used for all animals involved in the study.**

|  | **Recommendation** | **Section/Paragraph** |
| --- | --- | --- |
| **If humane endpoints* were used, report the following:** | | |
| **1** | **The specific criteria used to determine when animals should be euthanized** | **Materials and Methods/Surgical Technique** |
| **2** | **Once animals reached endpoint criteria, the amount of time elapsed before euthanasia** | **Materials and Methods/Surgical Technique** |
| **3** | **Whether any animals died before meeting criteria for euthanasia** | **Materials and Methods/Surgical Technique** |
| **If humane endpoints* were not used, report the following:** | | |
| **1** | **A scientific and ethical justification for the study design, including the reasons why humane endpoints could not be used, and discussion of alternatives that were considered but could not be used** |  |
| **2** | **Whether the institutional animal ethics committee specifically reviewed and approved the anticipated mortality in the study design** |  |

**ITEM 2.** **Include the following details of the study design and outcomes.**

|  | **Recommendation** | **Section/Paragraph** |
| --- | --- | --- |
| **1** | **The duration of the experiment** | **Materials and Methods/Surgical Technique** |
| **2** | **The numbers of animals used, euthanized, and found dead (if any); the cause of death for all animals** | **Materials and Methods/Surgical Technique** |
| **3** | **How frequently animal health and behavior were monitored** | **Materials and Methods/Surgical Technique** |
| **4** | **All animal welfare considerations taken, including efforts to minimize suffering and distress, use of analgesics or anaesthetics, or special housing conditions** | **Materials and Methods/Surgical Technique** |
| **5** | **Any special training in animal care or handling provided for research staff** | **Materials and Methods/Surgical Technique** |

***Definition of a humane endpoint**

A humane endpoint is an experimental endpoint at which animals are euthanized when they display early markers associated with death or poor prognosis of quality of life, or specific signs of severe suffering or distress. Humane endpoints are used as an alternative to allowing such conditions to continue or progress to death following the experimental intervention (“death as an endpoint”), or only euthanizing animals at the end of an experiment. Before a study begins, researchers define the practical observations or measurements that will be used during the study to recognize a humane endpoint, based on anticipated clinical, physiological, and behavioral signs. These may include, for instance, body temperature or weight changes, tumor size or appearance, abnormal behaviors, pathological changes, ruffled fur, reduced mobility, body posture, or expression of specific body fluid markers. Please see the NC3Rs guidelines for more information.

**ARRIVE Guidelines**

*PLOS ONE* encourages authors to follow the [Animal Research: Reporting of In Vivo Experiments (ARRIVE) guidelines](http://www.nc3rs.org.uk/arrive-guidelines) for all submissions describing laboratory-based animal research and to upload a completed [ARRIVE Guidelines Checklist](http://www.nc3rs.org.uk/sites/default/files/documents/Guidelines/NC3Rs%20ARRIVE%20Guidelines%20Checklist%20%28fillable%29.pdf) to be published as supporting information.
