# Supplementary material for: Differential gene analysis during the development of obliterative bronchiolitis in a murine orthotopic lung transplantation model: A comprehensive transcriptome-based analysis
Source: PLoS One. 2020 May 8;15(5):e0232884. doi: 10.1371/journal.pone.0232884 (PMC7209239; doi:10.1371/journal.pone.0232884)
Supplement: S1 Fig — To identify regulated transcripts in the data processing, the fold changes between the compared groups and P values with t test were calculated. The t test was performed on normalized data processed with log2 transforming. The threshold for selecting regulated transcripts was set at a ≥ 2-fold or ≤0.5-fold change and a P value <0.05 from sham group at mean value. For compared to non-OB, upregulated transcripts were extracted with P<0.05 among those 2,169 upregulated transcripts. (PDF) [file pone.0232884.s003.pdf]

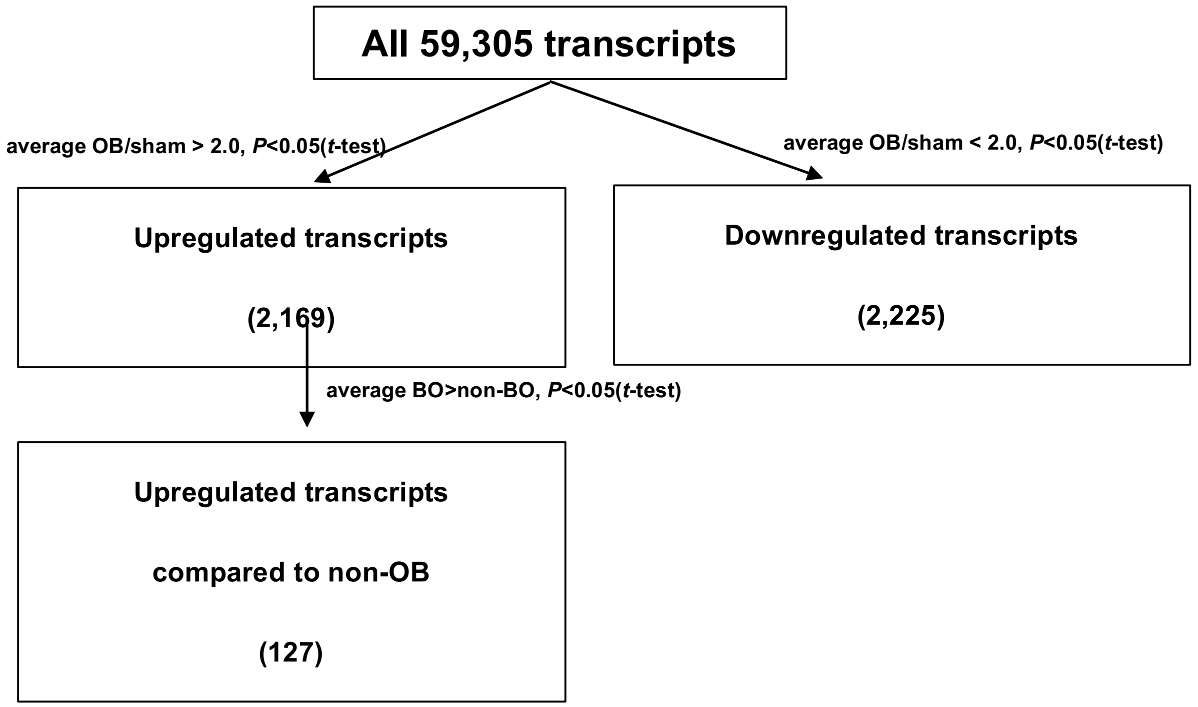

S1 Fig. Marker Extraction of BO. To identify regulated transcripts in the data processing, the fold changes between the compared groups and *P* values with *t* test were calculated. The *t* test was performed on normalized data processed with log2 transforming. The threshold for selecting regulated transcripts was set at a ≥ 2-fold or ≤0.5-fold change and a *P* value <0.05 from sham group at mean value. For compared to non-OB, upregulated transcripts were extracted with *P*<0.05 among those 2,164 upregulated transcripts.
