# Supplementary material for: Differential gene analysis during the development of obliterative bronchiolitis in a murine orthotopic lung transplantation model: A comprehensive transcriptome-based analysis
Source: PLoS One. 2020 May 8;15(5):e0232884. doi: 10.1371/journal.pone.0232884 (PMC7209239; doi:10.1371/journal.pone.0232884)
Supplement: S2 Fig — Unsupervised two-way hierarchical clustering based on standard deviation (above 1.8) classified lung samples into two groups: allografts and sham group. The two types of sham, C57BL/10 and C57BL/6, had similar mRNA expression compared to allografts. (PDF) [file pone.0232884.s004.pdf]

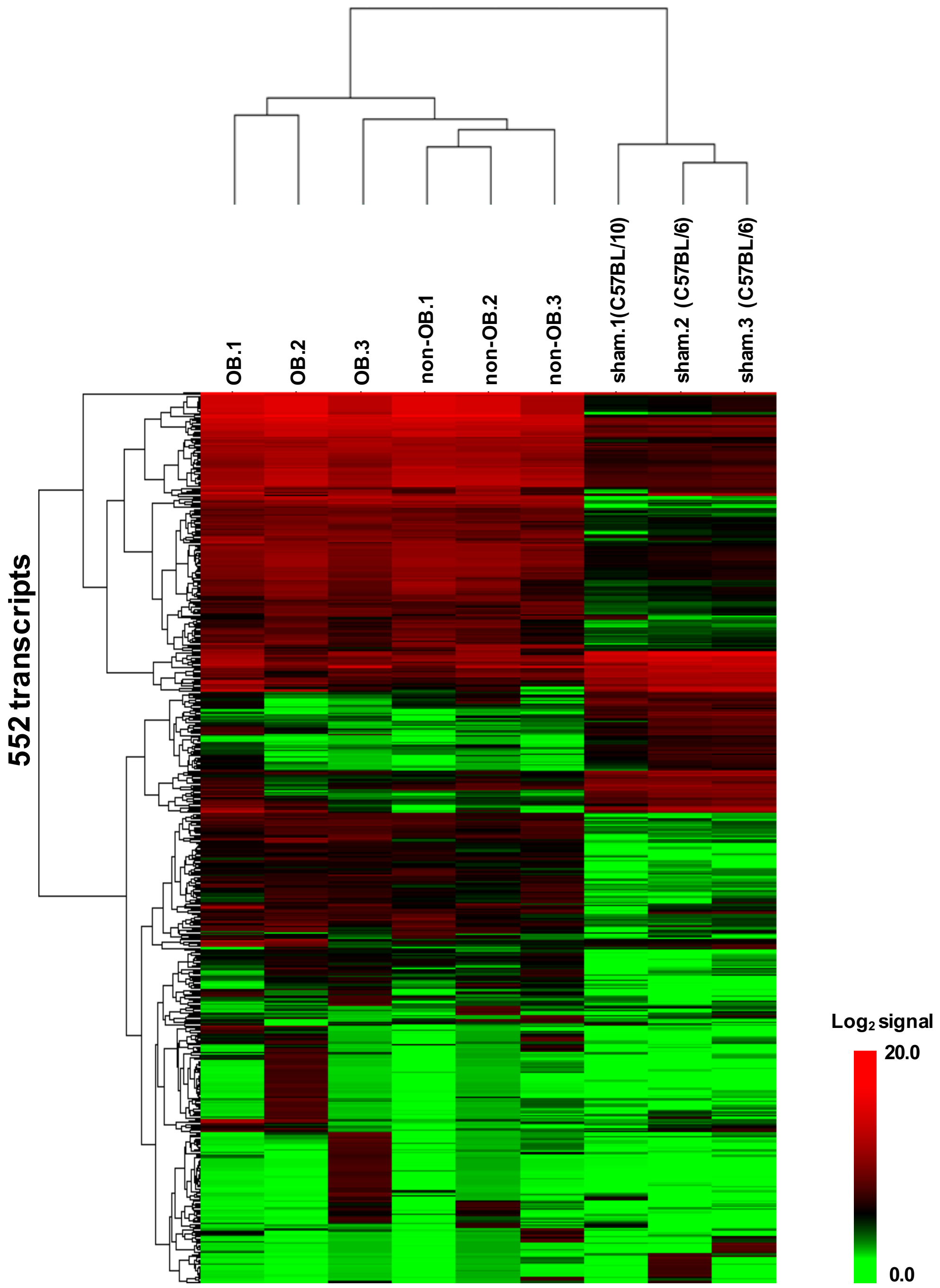

S2 Fig. Unsupervised two-way hierarchical clustering of all six samples.

Unsupervised two-way hierarchical clustering based on standard deviation (above 1.8) classified lung samples into two groups: allografts and sham group. The two types of sham, C57BL/10 and C57BL/6, had similar mRNA expression compared to allografts.
